# Rationale for combined therapies in severe-to-critical COVID-19 patients

**DOI:** 10.3389/fimmu.2023.1232472

**Published:** 2023-09-11

**Authors:** Aitor Gonzaga, Etelvina Andreu, Luis M. Hernández-Blasco, Rut Meseguer, Karima Al-Akioui-Sanz, Bárbara Soria-Juan, Jose Carlos Sanjuan-Gimenez, Cristina Ferreras, Juan R. Tejedo, Guillermo Lopez-Lluch, Rosa Goterris, Loreto Maciá, Jose M. Sempere-Ortells, Abdelkrim Hmadcha, Alberto Borobia, Jose L. Vicario, Ana Bonora, Cristobal Aguilar-Gallardo, Jose L. Poveda, Cristina Arbona, Cristina Alenda, Fabian Tarín, Francisco M. Marco, Esperanza Merino, Francisco Jaime, José Ferreres, Juan Carlos Figueira, Carlos Cañada-Illana, Sergio Querol, Manuel Guerreiro, Cristina Eguizabal, Alejandro Martín-Quirós, Ángel Robles-Marhuenda, Antonio Pérez-Martínez, Carlos Solano, Bernat Soria

**Affiliations:** ^1^ Alicante Institute for Health and Biomedical Research (ISABIAL), Alicante, Spain; ^2^ Institute of Bioengineering, Miguel Hernández University, Elche, Spain; ^3^ Applied Physics Department, Miguel Hernández University, Elche, Spain; ^4^ Clinic University Hospital, Fundación para la Investigación del Hospital Clínico de la Comunidad Valenciana (INCLIVA) Health Research Institute, Valencia, Spain; ^5^ Hospital La Paz Institute for Health Research, IdiPAZ, University Hospital La Paz, Madrid, Spain; ^6^ Réseau Hospitalier Neuchâtelois, Hôpital Pourtalès, Neuchâtel, Switzerland; ^7^ Department of Molecular Biology and Biochemical Engineering, University Pablo de Olavide, Seville, Spain; ^8^ Biomedical Research Network for Diabetes and Related Metabolic Diseases-Centro de Investigación Biomédica en Red de Diabetes y Enfermedades Metabólicas Asociadas (CIBERDEM) of the Carlos III Health Institute (ISCIII), Madrid, Spain; ^9^ University Pablo de Olavide, Centro Andaluz de Biología del Desarrollo - Consejo Superior de Investigaciones Científicas (CABD-CSIC), Centro de Investigación Biomédica en Red de Enfermedades Raras (CIBERER), Sevilla, Spain; ^10^ Nursing Department, University of Alicante, Alicante, Spain; ^11^ Biotechnology Department, University of Alicante, Alicante, Spain; ^12^ Biosanitary Research Institute (IIB-VIU), Valencian International University (VIU), Valencia, Spain; ^13^ Clinical Pharmacology Department, La Paz University Hospital, School of Medicine, Universidad Autónoma de Madrid, IdiPAz, Madrid, Spain; ^14^ Transfusion Center of the Autonomous Community of Madrid, Madrid, Spain; ^15^ Health Research Institute Hospital La Fe, Valencia, Spain; ^16^ Valencian Community Blood Transfusion Center, Valencia, Spain; ^17^ Immunology Department, Dr. Balmis General University Hospital, Alicante, Spain; ^18^ Department of Clinical Medicine, Miguel Hernández University, Elche, Spain; ^19^ Infectious Diseases Unit, Dr. Balmis General University Hospital, Alicante, Spain; ^20^ Intensive Care Service, Hospital Clinico Universitario, Fundación para la Investigación del Hospital Clínico de la Comunidad Valenciana (INCLIVA), Valencia, Spain; ^21^ Intensive Care Unit, Hospital Universitario La Paz – IdiPAZ, Madrid, Spain; ^22^ Emergency Department, Hospital Universitario La Paz – IdiPAZ, Madrid, Spain; ^23^ Banc de Sang I Teixits, Barcelona, Spain; ^24^ Department of Hematology, Hospital Universitario y Politécnico La Fe, Valencia, Spain; ^25^ Research Unit, Basque Center for Blood Transfusion and Human Tissues, Galdakao, Spain; ^26^ Cell Therapy, Stem Cells and Tissues Group, Biocruces Bizkaia Health Research Institute, Barakaldo, Spain; ^27^ Internal Medicine Department, Hospital Universitario La Paz – IdiPAZ, Madrid, Spain; ^28^ Department of Pediatrics, Faculty of Medicine, Universidad Autónoma de Madrid, Madrid, Spain; ^29^ Hematology Service, Hospital Clínico Universitario, Fundación para la Investigación del Hospital Clínico de la Comunidad Valenciana (INCLIVA), Valencia, Spain

**Keywords:** COVID-19, cytokine storm, immunomodulation, mesenchymal stromal cells, SARS-CoV-2, advanced therapies

## Abstract

An unprecedented global social and economic impact as well as a significant number of fatalities have been brought on by the coronavirus disease 2019 (COVID-19), produced by the severe acute respiratory syndrome coronavirus 2 (SARS-CoV-2). Acute SARS-CoV-2 infection can, in certain situations, cause immunological abnormalities, leading to an anomalous innate and adaptive immune response. While most patients only experience mild symptoms and recover without the need for mechanical ventilation, a substantial percentage of those who are affected develop severe respiratory illness, which can be fatal. The absence of effective therapies when disease progresses to a very severe condition coupled with the incomplete understanding of COVID-19’s pathogenesis triggers the need to develop innovative therapeutic approaches for patients at high risk of mortality. As a result, we investigate the potential contribution of promising combinatorial cell therapy to prevent death in critical patients.

## 1 Introduction

Severe acute respiratory syndrome coronavirus 2, or SARS-Cov-2, is a new coronavirus originally discovered after an outbreak of respiratory illness called COVID-19 in the Chinese city of Wuhan, Hubei (1). The infection of this virus quickly transitioned from being an isolated epidemic in a Chinese region to becoming a global health emergency of global alarm, and eventually a worldwide pandemic, due to the accelerated number of infections and fatalities that happened first in China and afterwards over the world. On March 11, 2020, given the rapid and progressive expansion of the epidemic internationally, the World Health Organization (WHO) declared a state of pandemic.

Coronaviruses belong to a broad virus family and happen to be the primary cause of common cold and some other diseases such as Middle East respiratory syndrome (MERS-CoV) and severe acute respiratory syndrome (SARS-CoV) (2, 3). They are encapsulated, and exhibit a spherical shape with the longest single-strand positive-sense RNA genome amid RNA viruses (4). Their name is due to a spike protein in their surface that resembles a crown.

SARS-CoV-2 is mostly spread through respiratory particles that are emitted when an infected individual sneezes, speaks or coughs. These particles, both small and large in size, tend to be concentrated within a short distance, the probability of transmission decreases with barrier methods as masks, physical separation and increased ventilation (5, 6).

**Figure 1:**
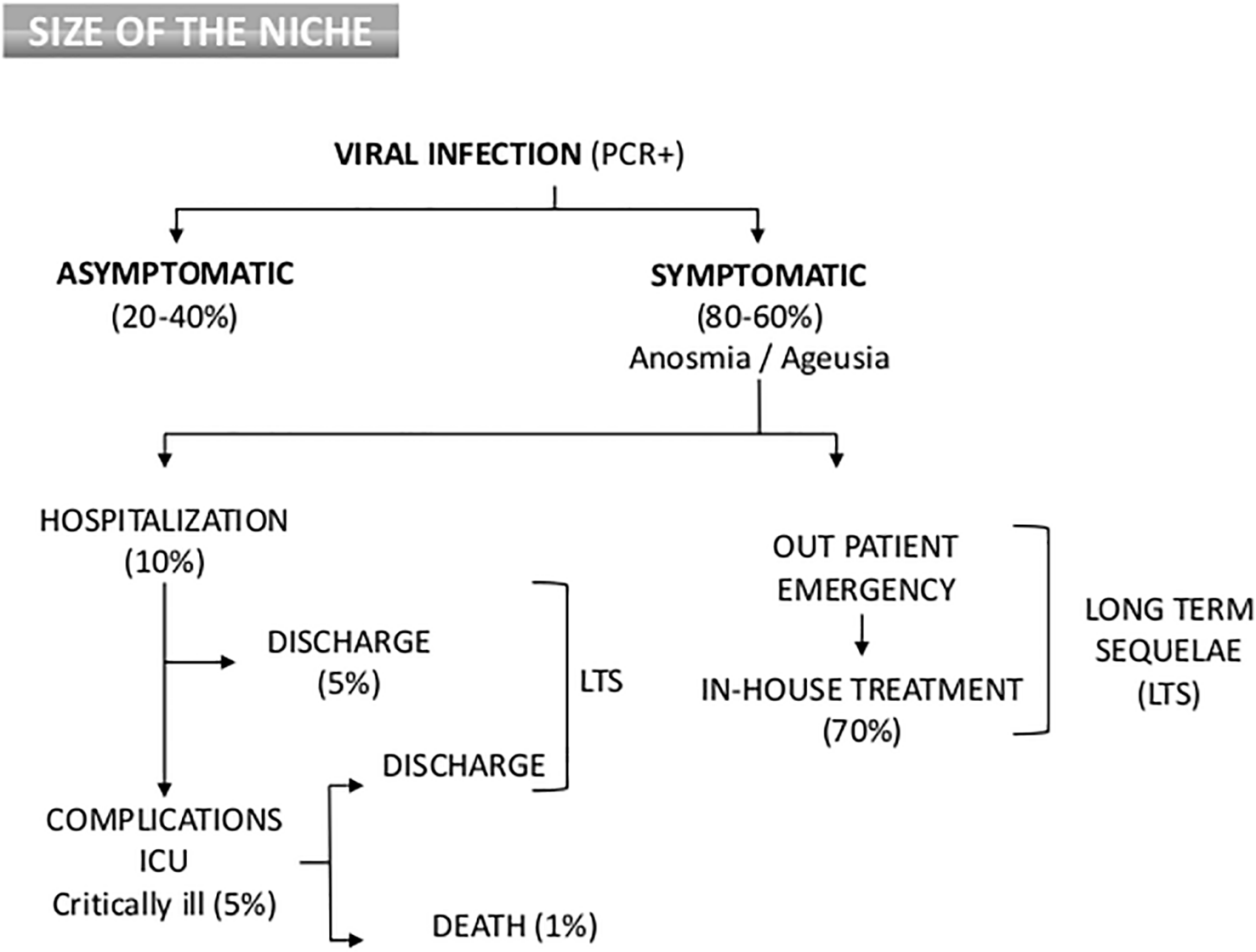
Flow Chart of clinical outcome and epidemiology distribution of COVID-19 patients. Percentages are dependent on variants, country, age and other morbidities.

Initial studies showed that SARS-CoV-2 infection can manifest across a wide clinical spectrum, ranging from asymptomatic infection to critical illness; for individuals who experience symptoms, the median incubation period of SARS-CoV-2 infection is typically around 4 to 5 days, and within an 11-day timeframe following infection, approximately 97% of individuals are likely to experience symptoms (7). Obesity, cardiovascular disease, chronic lung disease, diabetes, and advanced age are among the main risk factors associated with the progression of severe COVID-19 (6, 8, 9). Depending on the degree of severity of the clinical manifestations, the picture ranges from medium/moderate to severe and critical (**Table 1**). Clinical or radiographic evidence of lower respiratory tract disease, combined with a blood oxygen saturation level of 94% or above while the patient is breathing ambient air, indicates moderate disease. Severe disease can be identified by lung infiltrates (affecting more than 50% of the lung field within 24 to 48 hours), tachypnea (respiratory rate of 30 breaths per minute), hypoxemia (oxygen saturation of 93% or lower; ratio of partial pressure of arterial oxygen to fraction of inspired oxygen of 300), and tachycardia (heart rate exceeding 100 beats per minute) (6, 10) and patients can progress to Adult Respiratory Distress Syndrome (ARDS), which is a common immunopathological feature of severe COVID-19, SARS-1-CoV and MERS-CoV and is caused by an aggressive inflammatory response that can lead to respiratory difficulties and death (**Figure 1**).

**Table 1:** Clinical spectrum of infection severity.

Mild to moderate	Severe to critical
Fever	Immune Dysfunction
Dry cough	Lymphopenia
Smell/taste loss	Sustained inflammation
Headache	Secondary bacterial/fungi infection
Dizziness	ARDS (Acute respiratory distress syndrome
Nausea	Thrombosis
Diarrhea	Multiorganic damage (liver, kidney, myocardial)
Muscle/Joint pain Respiratory distress	

Throughout initial phases of the pandemic, approximately 17% to 35% of COVID-19 hospitalized patients were treated in an Intensive Care Unit (ICU), most likely due to hypoxemic respiratory worsening (11). Typically, children experience less severe symptoms primarily affecting the upper respiratory system, and hospitalization is rarely necessary, accounting for 2% to 5% of individuals who have been confirmed to have COVID-19 through laboratory testing exhibit an unclear susceptibility to the virus, as it is not well understood why they are less prone to contracting it, plausible causes include partial immunity from preceding viral exposures, lower exposure rates, and less strong immune responses, such as no cytokine storm (CS). Though the large percentage of pediatric cases are mild, a small proportion (7%) of hospitalized children, develop severe disease that requires mechanical ventilation (12).

## 2 High mortality risk COVID-19 patients: size of the niche

The evaluation of COVID-19 is based upon disease severity. Although severe sickness can affect anyone, most individuals with serious illness have at least one risk factor, several comorbidities and underlying diseases that eventually will progress into admission to the ICU, intubation, mechanical ventilation and death). Elder patients with comorbidities typically have severe to critical COVID-19 (13, 14). In a report from the Chinese Centre for Disease Control and Prevention (15), rates of case fatalities were respectively, 8% for those who age 70 to 79 year old and 15% for those aged 80 or older. This data contrasts to the 2.3% overall cohort case fatality rate (10), indicating that while symptoms of infection in kids and teenagers are often moderate, a tiny percentage do have severe and even fatal illness (16).

In a study of approximately 300,000 confirmed COVID-19 cases recorded in the United States, patients with documented co-morbidities exhibited a mortality rate that was 12-fold higher compared to those without such underlying conditions (17). Furthermore, the COVID-19 vaccine significantly decreases the risk of developing a severe disease and is linked to a lower fatality rate. Roughly, clinical features and distribution of COVID-19 population can be seen on **Figure 1**. Since a high proportion of SARS-CoV-2 infections are asymptomatic and mild infections go mostly undetected (18), the infection mortality rate has been estimated in many studies to be between 0.15% and 1%, with substantial variation by region and among different risk categories (19, 20).

As of May 31^st^ 2023, 689,549,946 people have been diagnosed with COVID-19, either by PCR assessment or any other available assay. Total death reported rises up to 6,884,636, close to 1% (21).

The most prevalent clinical signs of COVID-19 include dyspnea, dry cough, and fever. Typically, 2 days to 2 weeks after viral contact, symptoms start to appear (22). Previous studies conducted on 181 COVID-19 confirmed cases outside Wuhan, China showed that 5 days after initial exposure, symptoms started to occur, and 97% of people began to experience symptoms 11 days after infection (7). Additional signs experienced by some patients include ageusia, anosmia, myalgia, sore throat, fatigue, diarrhea, and headache (23, 24). While patients may initially present with chills and respiratory symptoms without fever, in later stages of the disease, dyspnea can progress to ARDS or multiple organ failure (8).

Since the initial phases of the pandemic, it was clear that the diversity in symptoms, age, genetics, geographic location and morbidity play dissimilar roles in viral transmission and disease spectrum. Complex biochemical and immunological studies are required to understand the genetic implications underlying severe COVID-19. Similar to other RNA viruses, SARS-CoV-2 undergoes continuous evolution through random mutations and therefore, those new mutations could affect its infectivity and virulence. Moreover, the ability of the virus to elude adaptive immune responses from previous SARS-CoV-2 infections, specific antivirals and antibodies may also amend the efficacy of vaccines or increase reinfection risk (25).

According to seroprevalence surveys (26–28), more than 33%, and even more than 50% of the global population will be infected with SARS-CoV-2 by 2022 (29, 30). With so many people still infected, understanding the duration and effectiveness of immunity is crucial for future considerations regarding protection against reinfections and severe disease.

Several variants of SARS-CoV-2 mutants, which display certain characteristics, such as increased virulence or transmissibility, have been acknowledged so far, Alpha (B.1.1.7), which was first seen in the United Kingdom, Beta (B.1.351), initially discovered in South Africa; Gamma (P.1), discovered in Manaus, Brazil; and Delta, first discovered in India and became dominant in July 2021 (31, 32).

In November 2021, the Omicron (B.1.1.529) variant was classified as a Variant of Concern (VOC) and rapidly emerged as the predominant variant worldwide. This VOC is more contagious than previous variants which, in fact, have largely disappeared worldwide (33). The clinical relevance, emergence and transmission of these new variants evolves quickly, especially for how it may affect the effectiveness of vaccines and the rates of transmission as well as effectiveness of current therapeutics (34). With the introduction of the Omicron variant, the fraction of completely asymptomatic cases may increase even further. Due to its increased transmissibility and the occurrence of steep epidemic waves, it is anticipated that the number of infected individuals will rise significantly with the emergence of the Omicron variant (35, 36).

Moreover, those individuals with a higher death rate (WHO grade 8), are typically male, older than 65, and show concomitant comorbidities such as obesity, high blood pressure, cardiovascular illnesses, cancer, type 2 diabetes, smokers, etc. Characteristics that can be used as early warning signs of a High Mortality Risk (37), as well as non-vaccinated people or immunocompromised patients that might result in a poor response to the COVID-19 vaccination. Even more, a previous study has identified three phenotypes among COVID-19 patients who were admitted to the hospital based on their age and sex, underlying medical conditions, clinical and laboratory data, as well as radiological features at the time of presentation. The phenotypes have therapeutic implications despite not being intended for use in mortality prediction, and relationships with patient prognosis were identified, leading to the development of a simplified probabilistic model that may be relevant to other cohorts. Phenotype A was typical for young women with mild respiratory symptoms and normal inflammatory parameters, phenotype B included mainly obese patients, lymphopenia and moderately high inflammatory values. Finally, phenotype C patients were older, with higher inflammatory parameters and more comorbidities (38). Furthermore, the International Severe Acute Respiratory and Emerging Infection Consortium (ISARIC) has published an assessment of patients’ mortality risk based on a cohort of more than 35.000 patients (39), with 8 elements readily available at admission (age, respiratory rate, peripheral oxygen saturation, sex, level of consciousness, number of comorbidities, C reactive protein and urea level).

### 2.1 SARS-CoV-2 virus entry and effects

Coronaviruses have an enveloped, positive sense single-stranded RNA genome. The five main proteins are the spike protein (S), membrane glycoprotein (M), an additional membrane glycoprotein (HE), nucleocapsid protein (N), and a small membrane protein (SM). The S protein belongs to the group I fusion glycoproteins. It has a homotrimeric structure with a single top conformation and two lower conformations. The spike is made up of two subunits, the N′-terminal S1 and the C′-terminal S2, which are each in charge of a specific function, such as interacting with the host cell or engulfing a virion, respectively. RNA genome is hold within the nucleocapsid while the S-protein, membrane proteins and envelope constitute together the viral envelope (40).

**Figure 2:**
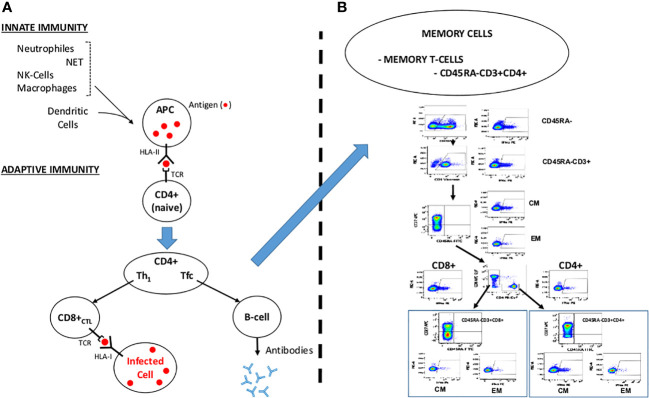
Overview of the Immune System Response. NET: Neutrophile Extracellular Traps. APC: Antigen Presenting Cell. NK: Natural Killer. TCR: T-cell Receptor. HLA-I/HLA-II: Human Leukocyte Antigen I and II. Th1: T Helper 1 Lymphocyte. Tfc: Follicular T Helper Cell. CD8+ CTL: Cytotoxic T Lymphocyte. **(A)** Course for a normal immune response engulfing innate and adaptive immunity. **(B)** Memory T-cells (modified from Ferreras et al, 2021). CD45RA-: CD45RA isoform depleted cells. CM: Central Memory T-cells. EM: Effector Memory T-cells.

Coronavirus spike proteins are class I fusion proteins that resemble the envelopes of other viruses such as human immunodeficiency virus (HIV) or hemagglutinin of influenza species (41). The SARS-CoV-2 virus uses the angiotensin-converting enzyme II (ACE2) receptor as a cellular entry (42), which is widely distributed in type II alveolar cells and as well as a small proportion of monocytes and macrophages, the capillary endothelium of the lungs and many other organs, including the hepatic, cardiovascular, gastrointestinal, and renal systems which also express ACE2 (43). ACE2 variant N720D may also enhance SARS-CoV-2 infectivity (44), and show more affinity of Furin to the mutated D614G S-protein of the virus (45). As soon as the viral spike protein attaches to the ACE2 receptor of the host cell membrane, the virus enters into host cells. Changes on the S-protein after docking grants the virus entrance to the endosomal pathway, virus RNA is then translated into viral components (46) in such a way that structural proteins are produced by the infected cell and their fragments exposed extracellularly by the Human Leukocyte Antigen (HLA)-II to the Antigen Presenting Cell (APC) (**Figure 2**).

### 2.2 Dysfunctional immune response

In the case of SARS-CoV-2 infection, severe disease is partially generated by the trigger of an unbalanced immune response. Innate immune cells must identify the virus’s invasion in order to stop the viral attack. This is done by pathogen-associated molecular patterns (PAMPs). Following the identification of viral genetic material, type I interferon (IFN) production is stimulated, and its signaling cascade activates important genes to inhibit viral replication and the development of a potent adaptive immune response (**Figure 2A**).

After an infection occurs, nasal epithelial cells increase the production of secreted immunoglobulins. In cases of severe COVID-19 infection, this natural mucosal defense mechanism can be exploited to harm the host by boosting the expression of pro-inflammatory cytokines (CS). Viral infections stimulate the enhancement of IFN regulatory genes (IRF3 and IRF7), which subsequently elevate the synthesis of type I IFN (47), in fact, nasal epithelial cells exhibit robust upregulation of both interferon I and III as their primary antiviral reaction (48). Type I IFNs play a vital role in regulating viral replication and bolstering the innate immune response (49).

The first barrier, the innate immunity, for any infection includes macrophages, neutrophils and NK-cells, mostly on the oropharyngeal mucosa. In 60-80% of the cases (asymptomatic) (18, 50–56), this barrier stops the virus and kills the virion-infected cell transmitting the message to the adaptive immune system. The HLA-II molecules of the antigen-presenting cells (APCs, mostly macrophages and dendritic cells) presents to the lymphocyte T-receptor (TCR) of the CD4-helper cells the antigens (“peptides” product of the hydrolysis of the S, M and N proteins) and activate into T-helper cells (Th): Th1 (in peripheral tissues) and follicular T-helper cells (Tfc, secondary lymphoid organs) which will, in turn, activate both the B-cells, which produce neutralizing antibodies and CD8^+^ cytotoxic cells, that kill virion infected cells. In convalescent patients recovered from COVID-19, memory NK-cells and B-cells remain in the body (in peripheral blood and lymphoid organs) ready to respond when these “foreign” antigens enter into the body (57).

Following the attachment of SARS-CoV-2 to the target cell, there is a possibility of over activation of the innate immune system and its associated cells, such as dendritic cells, macrophages, and granulocytes. The humoral and cellular immune system is subsequently activated by the complex of pro-inflammatory cytokines secreted by these cells. The immune system overreacts as a result of B-cell activation and antibody hypersecretion, causing tissue damage. Cytokines secreted from macrophages also recruit neutrophils and monocytes that penetrate the infection site too severely, damaging lung tissue and aggravating clinical symptoms. **Figure 2** summarizes a simplified example of the physiological response of the immune system. Memory T-cells can be subdivided into Central memory T-Cells CDRA-CCR7+, characteristic of the homing of T cells to the lymph nodes and mucosal tissues and effector memory T-Cells CD45RA-CCR7- that migrate to peripheral tissues. Our group has previously identified SARS-Cov-2 specific memory T-Cells, **Figure 2B**, modified from Ferreras et al. (58).

**Figure 3:**
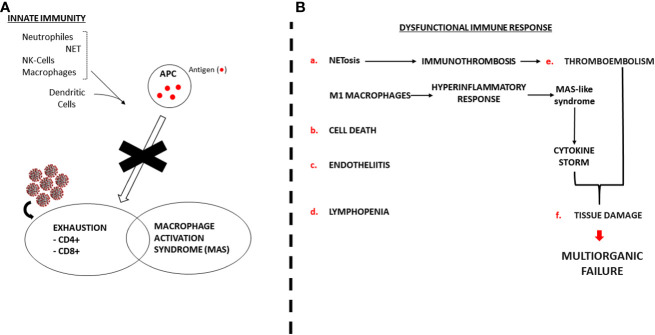
Dysfunctional SARS-Cov-2 induced immune response. NET: Neutrophile Extracellular Traps. APC: Antigen Presenting Cell. MAS: Macrophage Activation Syndrome. NK: Natural Killer. **(A)** SARS-Cov-2 blocks normal immune response. **(B)** Pathogenic mechanism of the dysregulated immune response.

**Figure 3A** depicts the dysfunctional SARS-Cov-2 induced immune response with CD8+ and CD4+ exhaustion (lymphopenia) and Macrophage over activation (Tumour Necrosis Factor-α and CS). Other effects of the virus on the immune system, endothelia and tissue damage are thoroughly described.

#### 2.2.1 NETosis and pyroptosis

In addition to engulfing bacteria, producing reactive oxygen species, degranulating, and secreting antimicrobials, neutrophils can also destroy invading pathogens, including viruses, by forming Neutrophile Extracellular Traps (NETs). NETs are extracellular fiber networks made mostly of neutrophil DNA that attach to and eliminate extracellular pathogens with little harm to the host cell. By harming endothelium cells, one of SARS-CoV-2 primary targets, neutrophils can also contribute to systemic viral dissemination (59–62). T-helper (Th) 1 cells and intermediate CD14+, CD16+ monocytes produce proinflammatory cytokine profiles. This is followed by neutrophil and macrophage infiltration into lung tissue, resulting in a CS (63). Rapid activation of pathogenic Th1 cells induces secretion of proinflammatory cytokines, such as interleukin-6 (IL-6) and granulocyte-macrophage colony-stimulating factor (GM-CSF). GM-CSF also activates CD14^+^ CD16^+^ inflammatory monocytes, causing them to produce vast quantities of IL-6, Tumour Necrosis Factor-α (TNF-α), and other cytokines (64, 65). IL-6, which is primarily linked to macrophages and dendritic cells (DCs), is one of the deleterious cytokines in COVID-19 linked with severe clinical conditions (66). This affiliation not only highlights the significant role of APCs in the development of the infection but also the widespread involvement of the innate immune system (67).

Once SARS-CoV-2 has entered the cell, rapid viral replication may result in significant vascular leakage and endothelial and epithelial cell death, which in turn triggers the production of a large amount of proinflammatory cytokines and chemokines and, as a direct result, cell pyroptosis (68).

Pyroptosis, a extremely inflammatory and Caspase-1-dependent form of programmed cell death, is a common reaction to intracellular pathogen infection and is a component of the immune system’s fight against infection. Interleukin (IL)-1β, which is released during pyroptosis, is raised in SARS-CoV-2 patients (8). Along with its beneficial effects on immune cell migration to inflamed tissues, IL-1β also increases on SARS-Cov2 and is largely released by macrophages through apoptosis and pyroptosis. In addition to IL-1βs beneficial effects on initiating and maintaining inflammation, NLRP3-IL-1 signaling has also been revealed to play a role in the COVID-19 CS. IL-1β, primarily released by macrophages through apoptosis and pyroptosis is elevated during SARS-Cov2 infection. It contributes positively to the recruitment of immune cells to inflamed tissues. Additionally, IL-1β is involved in initiating and sustaining inflammation, it has been hypothesized that NLRP3-IL-1β signaling also plays a role in the development of CS observed in COVID-19 (69).

NLRP3 is an innate immune system component that behaves as a pattern recognition receptor (PRR) that acknowledges PAMPs. There are several studies that suggest that IL-1β may contribute to COVID-19 CS in coronavirus infections (70, 71). In addition, alveolar epithelial cells and macrophages recognize pathogen-associated molecular patterns (PAMPs), damage-associated molecular patterns (DAMPs), and antibody-secreting cell (ASC) oligomers. This recognition leads to the release of proinflammatory chemokines, interferons, and cytokines, which attract immune cells, particularly T-cells and monocytes, from the bloodstream into the lungs affected by the infection (72), which might clarify the lymphopenia detected in the majority of the patients with SARS-CoV-2 infection (73). Additionally, SARS-CoV-2 induced pyroptosis among lymphocytes and macrophages exacerbates the lymphopenia in most of the observed patients (68).

In addition, SARS-CoV-2 infection, cell pyroptosis, and the resulting hyperinflammation might well induce NETosis, a controlled cell death process caused by neutrophils releasing NETs (74) that substantially lure and kill microbes as a mechanism of the innate immune response (75). However, if NETosis becomes dysregulated as it happens in acute and chronic inflammatory diseases (76), it can contribute to the pathogenesis of sepsis and ARDS, with NETs exacerbating multiorgan failure, microthrombi and vascular tissue damage (77). Preceding studies have described in severe cases of COVID-19, pathogenic immunothrombosis resulting from a dysregulated NET formation (59). COVID-19-related ARDS has been found to be linked to increased NET formation, and constitutes a potential marker of disease severity (78, 79).

Furthermore, the transmission risk is highly determined by the viral load (80) and viral antigens may well lead to severe disease and therefore induce a tougher antibody response (81). Previous studies in mice have showed that viral exposure dose defines likelihood of SARS-CoV-1 infection (80, 82) and SARS-CoV-2 research in golden hamsters were indicative of dose-dependent infection (83) (**Figure 3**). However, it is important to note that although patients admitted to ICU may exhibit a slower decline in viral load, the peak viral load of SARS-CoV-2 typically occurs within 5-6 days after the onset of symptoms (84), and does not vary between patients with mild and severe disease (85). A possible explanation could be that antibodies might exacerbate disease severity by antibody-dependent enhancement (ADE) as it happened with SARS-CoV outbreak in 2002 (86). Still, there is no present evidence backing this for SARS-Cov-2 (87). Moreover, SARS-CoV-2 RNA has been found in patients’ samples up until death, suggesting a clear correlation between poor disease outcome and viral load persistence (15).

#### 2.2.2 Cell death and Sars-Cov-2

It is widely recognized that cellular apoptosis and autophagy are fundamental processes within cells, serving pivotal roles in upholding balance and influencing disease development (88). A growing body of research suggests that cell death and autophagy induced by coronaviruses could hold significance in virus infection and the development of disease. The occurrence of lymphopenia has been linked with various indicators of severity, including the presence of the death ligand, FasL (88). An increasing body of evidence highlights the dual nature of these processes in the context of viral infections (89, 90). On one side, these processes hinder the virus’s replication and transmission by clearing infected cells through cell death. Conversely, dysregulated cell death leads to uncontrolled cellular damage and disruption in immune responses. Concurrently, viruses exploit cell autophagy to their advantage, utilizing it for replication niches, immune evasion, and extracellular release (91).

Infection with SARS-CoV-2 in lung epithelial cells triggers both cell death and an inflammatory response (92), to counteract this phenomenon, the application of caspase inhibitors can inhibit apoptosis, leading to the preservation of CD4+ T-cells and reverse the process of lymphocyte apoptosis (93).While early inflammatory responses are essential for constraining viral replication (94), coronaviruses have developed tactics to elude detection by the innate immune system by evading activation of pattern recognition receptors (PRRs) and disrupting downstream interferon (IFN) responses (95). Conversely, severe cases of COVID-19 are marked by an excessive inflammatory reaction both in the lungs and bloodstream (8, 96). In such cases, a blend of cytokines, especially TNF-α and interferon gamma (IFN-γ), trigger a form of inflammatory cell death termed PANoptosis, subsequently resulting in a CS (96, 97).

#### 2.2.3 Endotheliitis

Recent research indicates that endothelial damage and the resulting morphological and functional alterations in the endothelium are significant contributors to COVID-19-induced hyperinflammation. COVID-19 is expected to have a greater effect on the lungs, as they are the first organs to become infected and regenerate very slowly. Previous research suggest that SARS-CoV-2 infection causes a disproportionate immune response, known as a COVID-19 CS in severe COVID-19 cases (98, 99). Post-mortem histology from three patients with late-stage COVID-19 showed viral inclusions in microvascular lymphocytic endotheliitis and endothelial apoptotic cells, with infiltration of inflammatory cells around the vessels and endothelial cells, as well as signs of endothelial apoptotic cell death in the kidney, lung, heart and small bowel (100). Furthermore, recent research has found indications of endothelial glycocalyx disruption in the plasma and serum of 19 critically ill COVID-19 patients (101). This specific result is noteworthy because the integrity of the endothelial glycocalyx, which covers the luminal surface of endothelial cells, is essential for the preservation of vascular homeostasis (102). According to these results, SARS-CoV-2 infection has been observed to directly contribute to the development of endotheliitis in various organs. This involvement is evidenced by the presence of viral bodies within endothelial cells and the accompanying inflammatory response of the host. Additionally, in individuals with COVID-19, activation of apoptosis and pyroptosis may play a significant role in the damage to endothelial cells. The systemic decreased microcirculatory performance in various arterial beds and its clinical consequences in COVID-19 patients might be stated through COVID-19-endotheliitis (100).

The stabilization of the endothelium while preventing viral replication, primarily with anti-inflammatory anti-cytokine drugs, ACE inhibitors, and statins, this aspect of SARS-CoV-2 infection and the development of endotheliitis becomes particularly significant for vulnerable patients who already have pre-existing endothelial dysfunction. Factors such as male sex, hypertension, smoking, obesity, established cardiovascular disease, and diabetes, which are associated with endothelial dysfunction, are also known to be linked with unfavourable outcomes in COVID-19 (103–105).

#### 2.2.4 Lymphopenia

Previous clinical findings indicate that lymphocyte measurements including B cells, natural killer (NK), CD8+ cytotoxic T and CD4+ T cells were all reduced in COVID-19 patients (106, 107). A thorough study of B and T cell populations in COVID-19 patients found a link between increased disease severity and lower numbers of CD8+ and CD4+ T cells. Furthermore, the drop in CD8+ T cells was greater in cases with milder illness than the decrease in CD4+ T cells (108). Mechanisms involved in lymphocyte depletion induced by SARS-CoV-2 might be due to direct T-cell infection via ACE2 receptor, resulting in T-cell death (109). The depletion and exhaustion of T-cells with their respective function can be speeded by a number of pro-inflammatory or anti-inflammatory cytokines, and the virus may kill secondary lymphoid tissues like lymph nodes and the spleen (110).

A major contributing component to the observed lymphopenia is probably due to the inflammatory CS. TNF-α and IL-6 levels in the serum have been found to be strongly linked with lymphopenia, whereas levels in the serum of healed patients are nearly normal. Massive lymphocyte death was discovered during autopsy examinations on lymphoid organs taken from numerous individuals who passed away from the condition; this death was ascribed to high levels of IL-6. Tocilizumab, an IL-6 receptor antagonist, treatment increased the number of circulating lymphocytes, further indicating that an increase in IL-6 is a major factor in the development of lymphopenia (111). Even more, treatment of critical ICU patients with intubation, mechanical ventilation and extracorporeal membrane oxygenation (ECMO) with adipose-tissue derived Mesenchymal Stromal Cells (MSCs) increase CD4+, CD8+ and B-cells, suggesting that inflammation synergizes with other mechanisms causing lymphopenia (112). This sequence of inflammatory events originates from the liberation of pro-inflammatory cytokines, notably IL-18 and IL-1β. This phenomenon elucidates the notable feature of commonly observed neutrophilia and leukopenia (113). Overall, the harshness of the disease has been closely linked to lymphopenia and hyper-inflammatory response.

#### 2.2.5 Thromboembolism

Early findings indicated greater incidence of venous thromboembolism (VTE) in individuals with severe COVID-19 disease compared to data from similar patients not afflicted by SARS-CoV-2 in addition to respiratory problems. Patients with COVID-19 usually experience coagulation problems, and those with severe sickness frequently have high levels of coagulation markers such D-dimer and fibrinogen degradation products. Along with disseminated intravascular coagulation, severe thrombocytopenia and lymphopenia have also been linked to worse outcomes (114). As a result of the thromboinflammation brought on by COVID-19, medical research has also shown that patients with COVID-19 have an increased risk of developing pulmonary embolism (PE). In COVID-19, thromboinflammation appears as increased levels of procoagulants (like von Willebrand factor) and endothelial dysfunction, which reduces the endothelium’s protective antithrombotic action (115, 116).

#### 2.2.6 Tissue damage

It has also been shown that SARS-CoV-2 principally affects the lung by generating diffuse alveolar destruction with ARDS, yet the recent research has revealed that the virus also has a negative influence on further important organs and tissue, such as the heart, brain, large intestine, kidneys, and spleen (117–120). In COVID-19 patients, there is a link between inflammation and serious organ damage. Diffuse alveolar injury, including damage to hyaline membranes, is the main pathophysiology in ARDS. Pneumocytes’ viral cytopathic effect suggests that there has been direct viral harm (121).

Emerging evidence suggests that SARS-CoV-2 has the capability to efficiently propagate and replicate in various organs, including the lungs, brain, heart, spleen, liver, and gastrointestinal tract. This is supported by findings on the virus’s route of entry and distribution, as well as the presence of infective RNA in these organs. These findings indicate the need to explore alternative mechanisms of cellular entry, such as non-receptor mediated endocrine entry, in addition to the conventional understanding of cellular entry through the ACE2 receptor.

### 2.3 T-cell and B-cell disorders

The resolution of a viral infection encompasses both cellular and humoral immune reactions. The humoral immune response, too, assumes a vital role in eradicating the virus within the host. The antibody responses specific to SARS-CoV-2 exhibit differing levels and characteristics between individuals with asymptomatic infections and those experiencing severe disease.

B cells play a crucial role by producing antibodies and facilitating the humoral immune response, rendering them highly significant in establishing protective immunity against SARS-CoV-2 (122), in addition T cells differentiate into cytotoxic T lymphocytes (CD8+) and helper T-cells (CD4+) engaging with the COVID-19 infection through distinct interactions (123).

Sustained defense hinges on the expansion of memory T cells and B cells tailored to SARS-CoV-2. Notably, follicular T-helper cells drive the humoral immune reaction, fostering a reservoir of specialized memory B cells primed to respond swiftly if confronted with a potential reinfection (124). The cytotoxic program in CD8+ T cells persists, type 1 cytokines and interleukin-17 (IL-17) production rise in T cells from patients who are in the recovery phase. It’s interesting to note that B cells from people with acute COVID-19 showed an imbalance between the cytokines IL-6 and IL-10 in response to the activation of Toll-like receptors, leaning toward a pro-inflammatory profile. Regardless of the clinical results, the frequency of IL-6+ B cells reverted to normal in convalescent patients, but the recovery of IL-10+ B cells was associated with the resolution of lung problems (125).

## 3 COVID-19 time-course

**Figure 4:**
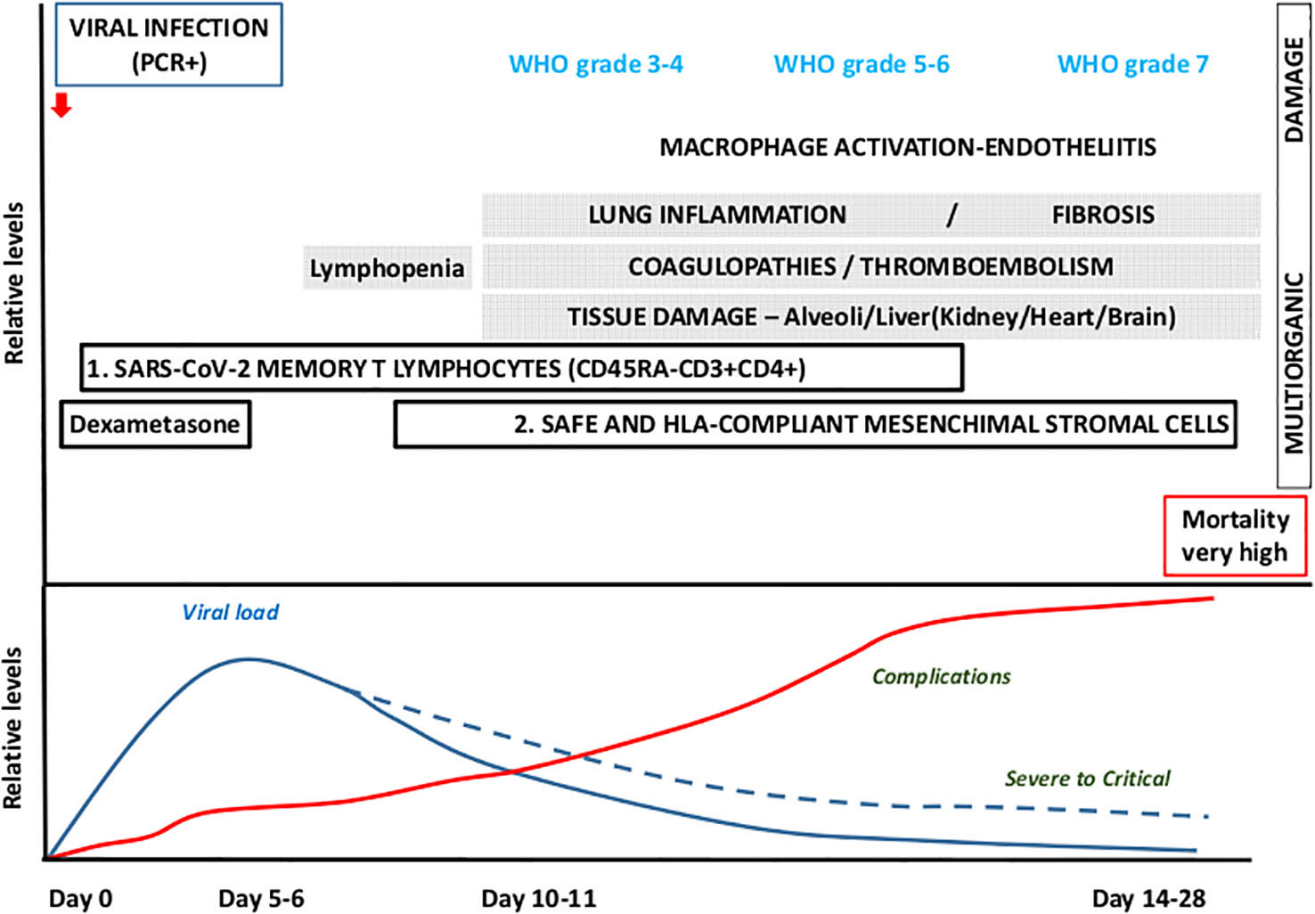
Time-course and clinical outcomes for COVID-19. Conservative treatment is being used for the first infection phase, which lasts up to 3–5 days and progresses with mild symptomatology and increased viral load, whereas in a small proportion of patients the second phase, 7 day onwards, yields extensive inflammatory response and an anti-inflammatory approach is recommended. Therapeutic proposal: Use SARS-Cov-2 memory CD45RA-CD3+CD4+ during the first phase (viral) and safe and HLA-compliant mesenchymal stromal cells for the second phase (complications).

COVID-19 shows several phases. During the early infection phase, usually mild symptomatology is reported such as, fever, cough, headache, etc. Laboratory abnormalities are lymphopenia, D fragments of the fibrin protein (D-dimer), low LDH, etc. It is also characterized by an increased viral load, which encourages a conservative treatment based on antiviral agents. In a second phase, the inflammatory response would predominate, leading to pulmonary inflammation or fibrosis, coagulopathies, and tissue damage and where anti-inflammatory drugs would have greater importance (**Figure 4**).

SARS-CoV-2 clearance from the lungs and upper respiratory tract should be effective in the majority of COVID-19 patients as a result of the first cytokine release, activation of the antiviral interferon response, and recruitment of immune cells; nevertheless, an abnormal immune response could lead to a severe disease outcome. Certainly, the excessive production of inflammatory cytokines and chemical mediators, along with macrophage activation, endotheliitis, lung hyper-inflammation, coagulopathies, thromboembolism, and tissue damage, collectively contribute to a life-threatening response. This response is widely regarded as the primary cause of the severity of COVID-19 and can lead to fatalities in affected patients (126, 127). Furthermore, these life-threatening responses are associated with elevated levels of circulating cytokines, severe lymphopenia, thrombosis, and extensive infiltration of mononuclear cells in multiple organs (73, 128, 129).

We could roughly discuss the physiopathology and viral characteristics as follows; early infection, in which a viral infection results in an initial immune system depression that frequently results in significant lymphopenia (130). The virus infects ACE-2 receptor-containing cells (endothelial, alveoli, gut epithelia, kidney, etc.), causing a systemic endothelial inflammation that manifests clinically as fever, dry cough, and lymphopenia. This inflammation is more severe in patients with prior chronic endotheliitis (old age, obesity, atherosclerosis, hypertension, diabetes, etc.). In around 10–20% of infections, the virus is not stopped by the body’s first line of defense (NK-cells, neutrophils, macrophages, IFN-γ, etc.), the viral load rises, and pneumonia with lymphopenia occurs, requiring hospitalization (**Figure 1**). A hyper-inflammatory picture that mimics Macrophage Activation Syndrome (MAS), Graft-versus-Host Syndrome (GvHD), or secondary hemophagocytic lymphohistiocytosis is present in hospitalized patients along with symptoms such as respiratory distress, fever, hypoxia, etc.

Thromboembolism, tissue damage, and inflammation, a very severe systemic inflammatory phase requiring an intensive care unit develops in about 5% of infected patients. Unlike the SARS-1 coronavirus, the host reaction is highly robust and varied. The development of a major inflammatory phase (131), which leads to ARDS, is in fact one of the most obvious features of the physiopathology of pneumonia in COVID-19 illness (132) and a condition that is similar to the MAS (126, 133) and injury to tissues that might possibly require ECMO. Additionally, a progressive endothelial thromboinflammatory syndrome (with raised D-dimers levels) uncommon in other viral infections, aggravates the disease’s prognosis (107, 134).

## 4 Combinational therapies

Along with vaccine research and other strategies that obstruct viral entry or directly target the virus, therapies that treat the immunopathology of the illness are increasingly being prioritized. The use of monoclonal antibodies such as Sotrovimab or Tixagevimab-cilgavimab for modulating the inflammatory response can constitute an alternative therapeutic strategy. IFN, IL-1, IL-6, and complement factor 5a are all mediators that target the extreme inflammatory response that occurs after SARS-CoV-2 infection with the aim of avoiding organ damage (135–137) thus monoclonal antibodies aiming to block these factors, together with tyrosine kinase inhibitors are being used to block the cytokine storm-like response or their ability to avert pulmonary vascular leakage in people with COVID-19.

### 4.1 Convalescent plasma from donors

The earliest reports of treatments using plasma from patients who had recovered from viral illnesses were published during the 1918 flu pandemic. Clinical improvement was seen in all participants both before and after receiving convalescent plasma transfusion status, according to a preliminary study of 5 critically sick COVID-19 patients treated with convalescent plasma encapsulating neutralizing antibodies (138). Nevertheless a multicenter randomized open-label clinical trial for convalescent plasma in patients hospitalized with COVID-19 pneumonia (139) did not result in a clear protection.

### 4.2 Cytokine inhibitors

Alongside with the clinical symptoms linked to viral invasion, the CS has attracted the most interest, raising the hypothesis that anti-inflammatory treatments aimed at lowering interleukin-6 (IL-6), IL-1, or even TNF-α may be helpful. It has been established that increased plasma levels of cytokines and chemokines, IL-6 in particular, are significantly higher in severe than in mild to moderate disease allowing to predict COVID-19 severity and survival (140–142) and correlate with the harshness of the disease. Early reports showed augmented plasma concentrations of IL-6, therefore providing the introduction of anti-IL-6 therapies in randomized clinical trials (143, 144) therefore, novel cytokine inhibitors, including Baricitinib, Anakinra, and Tocilizumab, were likely options for treating severe COVID-19 which found appropriate niches (145–147).

Baricitinib, an inhibitor of Janus-Associated Kinases (JAKs), which belong to a family of intracellular, non-receptor tyrosine kinases that transduce cytokine-mediated signals via the JAK-STAT pathway and since and JAKs mediate actions of many pro-inflammatory cytokines, combines both antiviral and anti-inflammatory effects. JAKs inhibitors block the signal transduction pathways that activate the immune system and cause inflammation, therefore, constitute an attractive treatment approach to stop the development of more serious disorders (148), considering the significant role that this transduction channel had in the onset of the CS in COVID-19 (149).

It is clear that therapeutic approaches targeting cytokine responses, in addition to anti-viral medications, deserve special attention to reduce morbidity and mortality in COVID-19 patients, even though the mechanisms by which SARS-CoV-2 infection induces cytokine overproduction are not yet fully understood (150, 151).

### 4.3 Corticosteroids, dexamethasone

Patients with autoimmune and inflammatory conditions such asthma, systemic lupus erythematous, Crohn’s disease, and rheumatoid arthritis are given prescriptions for glucocorticoids. Systemic corticosteroids (such as hydrocortisone, dexamethasone, and methylprednisolone) are given orally or intravenously and are effectively dispersed throughout the body, showing a variable degree of relative anti-inflammatory effectiveness and mineralocorticoid effects. However, high doses inhibit the immune response.

Dexamethasone, a synthetic corticosteroid that has the ability of dampening the immune system, which discouraged its use during early phases of COVID-19 infection. RECOVERY Trial showed a beneficial outcome using low dose (6 mg/day) in severe patients, defined as those with oxygen saturation lower than 94% (152). Dexamethasone reduced deaths by one-fifth in other hospitalized patients receiving only oxygen, but there was no benefit seen in COVID-19 patients who did not require respiratory support. Patients who had symptoms for more than 7 days and needed mechanical ventilation benefited the most. On the other hand, there was no benefit (and potential harm) amid patients who had shorter symptom duration and no need for supplementary oxygen. Furthermore, it could be extremely dangerous during recovery because not only will the virus persevere, but the body will be prevented from producing protective antibodies (153, 154).

In summary, numerous randomized trials have found that systemic corticosteroid therapy improves clinical outcomes and drops mortality in COVID-19 hospitalized patients who need supplementary oxygen (155, 156), perhaps by dampening the systemic inflammatory response induced by COVID-19, which can result in lung injury and multisystem organ dysfunction.

### 4.4 Cell-based therapies

#### 4.4.1 Memory T lymphocytes CD45RA-

The role of adaptive immunity in COVID-19 and the protective immunity conferred by T-cells and the role of memory T-cells in providing protection against SARS-CoV-2 has not yet been properly defined (58, 157, 158). Specific memory T-cells for other coronavirus has been found up to 11 years after infection (159), particularly in the context of allogeneic hematopoietic stem cell transplantation (HSCT). This immunological memory assigns a swift and fast secondary immune response that is decisive and forms the basis of adoptive cell treatment for viral infections in immunosuppressed patients. Infusion of CD45RA- memory T-cells reduces induced mortality and CD45RA- T-cells alloreactivity of GvHD in bone-marrow transplantation (160, 161).

**Figure 5:**
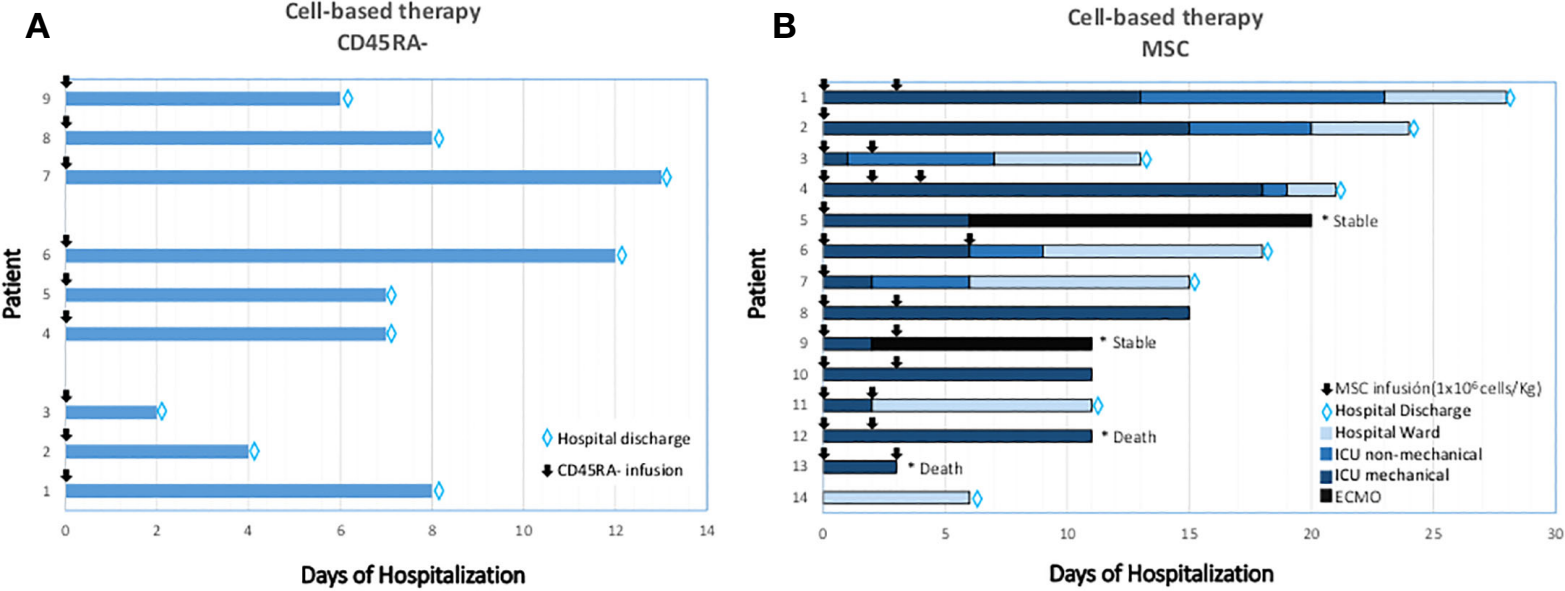
Clinical outcomes after CD45RA- **(A)** and MSC **(B)** treated patients. **(A)** T-cell memory based therapy (CD45RA depleted infused). Time-course and clinical outcome. **(B)** Cell-based therapy. Mesenchymal Stromal-stem Cell (MSC) infusions and timing are represented in arrows. In the X axis, days from the first MSC infusion are specified. Type of ventilation support is graded in colors through each row. MSC: Mesenchymal Stromal-stem Cells; ICU: Intensive Care Unit; ECMO: Extra Corporeal Membrane Oxygenation.

Previous research suggested that CD45RA- memory T-cells from the blood of convalescent donors include a SARS-CoV-2 specific T-cell population that is easy, efficient, and quick to isolate and subsequently, may be able to clear virally infected cells and confer T-cell immunity (**Figure 2**) (58). CD45RA- memory T cells from convalescent donors harboring SARS-CoV-2 specific T-cells were infused intravenously in a Phase 1 research clinical trial to assess the safety and feasibility of adoptive cell treatment for moderate to severe COVID-19 cases (NCT04578210). Nine patients with lymphopenia and pneumonia who had at least one HLA Class I match with the donor were enrolled (**Figure 5A**). The CD45RA- memory T cells were administered to the first 3 patients at a low dose (1x10^5^ cells/kg), the following 3 patients at an intermediate dose (5x10^5^ cells/kg), and the last 3 patients at a high dose (1x10^6^ cells/kg). There were no documented severe negative effects. Six days following the infusion, the patient’s clinical state improved as measured by the National Early Warning Score (NEWS) and a 7-category point ordinal. Following infusion, the median length of stay in the hospital was 8 days for the low dose group, 7 days for the intermediate dose group, and 4 days for the high dose group. Two weeks following the infusion, the inflammatory markers normalized and all displayed lymphocyte recovery. This study provides early evidence that the use of allogeneic CD45RA- memory T-cells from convalescent donors for the treatment of COVID-19 patients with moderate to severe symptoms is possible, safe, and related with rapid clinical improvement and brief hospital stays (162). Adoptive T-cell therapy has essentially no side effects, and the cell product can be preserved in a lymphocyte biobank to allow ready access in the event of future viral pandemics (58). Repeated infusion of CD45RA- cells from family donors have also been used to refractory viral and fungi infections in transplanted individuals (163).

#### 4.4.2 Mesenchymal stem-stromal cells

Advanced Therapy Medicinal Products (ATMPs) are cutting-edge therapies that include gene therapy, somatic cell therapy, and tissue-engineered products. Mesenchymal Stem-stromal cells (MSCs) are adult stem cells that have immunomodulatory, regenerative, and differentiation capabilities. Among them MSC have demonstrated to be safe in more than 400 clinical trials (Clinicaltrials.gov). As for October 31, 2022, MSC were proposed to be used in 37 clinical trials to treat COVID-19: 8 of them completed, 6 still recruiting, 7 active not recruiting, 4 suspended or withdrawn and the rest unknown.

MSC are multipotent non-hematopoietic progenitor cells with different degrees of stemness, derived from the mesodermal germ layer and resident in most of tissues (164). MSCs can be sourced from different types of tissues, including adipose tissue, Wharton’s jelly tissue, bone marrow, and amniotic fluid. They are able to differentiate in a wide range of cell types such as chondrocytes, osteocytes, neural cells, myocytes, and epithelial cells (165). The International Society for Cellular Therapy (ISCT) proposed the minimum criteria for the characterization of human MSC: (i) adherence to the plastic of the culture plate, (ii) adipogenic, chondrogenic and osteogenic differentiation capacity, and (iii) a specific profile of surface markers CD45+, CD90+ and CD73+, CD105+, HLADR-, CD11b- or CD14-, CD19- or CD79a- and CD34 (166, 167). Due to its unique biological properties including adhesion to plastic, easy expansion and culture, MSCs are the cell type mostly used in Cellular Therapy.

MSCs were first employed in humans as a cellular therapy in 1995, and they have since been used in basic research and clinical applications (168). The use of allogeneic MSCs in COVID-19 might prove useful due to its capabilities to diminish this clinical and biological picture of massive inflammation, and there are evidences of efficacy in GvHD (169, 170). Bone marrow-derived MSC appeared to be highly successful as a therapeutic option for ARDS on a phase I-II trial (171).

MSCs from the umbilical cord (UC-MSC) exhibit minimal immunogenicity and both immunoregulation and tissue-fixing abilities. They are an outstanding option for allogeneic adoptive transfer treatment because of this. It may be used to treat acute lung damage brought on by the H5N1 infection which shared a comparable inflammatory cytokine profile with COVID-19 (172–174). Mesenchymal Stromal-stem cell therapy might have the ability to avoid the release of cytokines via immune system, endorsing endogenous repair because of the regenerative properties of the stem cells. The MSC cells may aid in restoring lung microenvironment, protect alveolar epithelial cells, and treat pulmonary dysfunction and COVID-19 associated pneumonia (175). Furthermore, it has been shown that intravenous infusion UC-MSC as an adjuvant therapy to critically ill patients increases their chance of survival by inducing an anti-inflammatory state (176). The main reason for improvement of COVID-19 disease is the higher anti-inflammatory features of the MSCs after its intravenous provision. Furthermore, previous studies suggest that direct cell to cell mitochondrial transmission from MSCs to the alveolar epithelium and immune cells restricts the inflammatory response (177, 178). Among all stromal cells, Placenta-derived decidua stromal cells (DSCs) have been found to show a more robust immunosuppressive effect than other sources of MSCs. These DSCs display significantly stronger immunomodulatory and anti-inflammatory properties, making them successful in treating and managing steroid-refractory acute GvHD (179).

MSC therapy is thought to have the potential to prevent the excessive release of cytokines during an immune system response known as a cytokine storm. Additionally, MSCs possess reparative properties that can stimulate the body’s natural healing processes. These stem cells are capable of suppressing the proliferation of immune cells and modulating the functions of both innate and adaptive immune cells (180). Proinflammatory cytokines, such as TNF-α, IFN-γ, IL-1β, IL-2, IL-6, IL-8 and IL-17, signal over their receptors in MSC surface and stimulate biosynthesis of HGF, LIF, TSG-6, TGF-β, IL-10, and expression of superoxide dismutase (SOD), prostaglandin-E2 (PGE2), indoleamine-pyrrole 2,3-dioxygenase (IDO), nitric oxide synthase (iNOS, produced by murine cells) and cyclooxygenase-2 (COX-2) produced by human cells (181, 182). It has been shown that these molecules mediate the immunomodulatory and immunosuppressive properties of MSCs (180).

Our group was the first to use allogeneic MSC to treat critical mechanically ventilated COVID-19 patients (**Figure 5B**, Patients 1-13). Preliminary results of the BALMYS trial (NCT 04348461, suspended by lack of funding), indicate that critically ill patients with COVID-19 pneumonia can be safely administered with MSC derived from adipose tissue (AT-MSC) in and that administration was followed by clinical improvement and changes in inflammatory markers and recovery of immune populations (B-cells, CD4+ and CD8+ cells), these results suggest a potential biological effect of the cells. Only steroids were given concurrently when the cells were delivered. During their time in the ICU, the majority of patients received supportive care. This comprised normal treatments such as sedation and mechanical ventilation in addition to the use of vasopressors or inotropic medications, enteral or parenteral feeding, diuretics, and/or antibiotics. The findings imply that early adipose tissue-derived allogeneic MSC therapy during mechanical intubation may enhance the outcome (112). We also showed the absence of side effects following cell administration in these critically ill patients with respiratory failure, extensive inflammation, and prothrombotic risk. Allogeneic MSC was used to treat 13 adult COVID-19 patients who were receiving invasive mechanical ventilation and had previously taken antiviral and/or anti-inflammatory medications (including hydroxychloroquine, steroids, Tocilizumab, and/or Lopinavir/Ritonavir, among others) (112). Patient 14 was a Grade 4 (no-intubated, no-mechanical ventilation, that received bone-marrow derived mesenchymal stromal cells infused intravenously (183).

Nonetheless, pre-clinical evidence of the potential of MSCs is still limited and, even debatable (184, 185). MSCs are particularly useful because in addition of being anti-inflammatory and promoters of tissue regeneration they lacked ACE-2 Receptor (175) and the transmembrane protease serine 2 making them resistant to SARS-CoV-2 infection.

MSCs role plays through a paracrine mechanism, releasing biologically active substances known as the secretome (186) which is composed of soluble proteins, including a diverse range of growth factors, chemokines, and cytokines, as well as extracellular vesicles (EVs) (187) that are capable to interact with the target cells and modulate cellular responses. Because of its immunomodulatory, pro-angiogenic, anti-protease, regenerative, and anti-inflammatory properties, MSC-secretome could prove as a promising cell-free therapeutic tool both for acute and chronic lung diseases.

MSC therapies act by modulating responses in inflammatory diseases (188), migrate to sites of injury and inflammation in which they can exert their immunomodulatory effects, through a paracrine mode of action (189). Its immunomodulatory activity derives from their capability to *in-vitro* suppress proliferation and action of B, T and NK-cells, as well as dendritic cells, differentiation of monocytes to anti-inflammatory macrophages, differentiation of effector T cells to regulatory T cells and modulation of cytokine secretion (190).

Several clinical trials have assessed the numerous MSC sources used in COVID-19 to date (191, 192). MSC therapies have shown improved clinical symptomatology when comparing to conventional treatment (175, 193–195), and have also improved the survival of severe/critical patients even of those who developed ARDS (196–198).

One issue that must be taken into account is the proliferative activity of the MSC that might be affected by its origin as well as donor age (199, 200), therefore MSCs from older donors may have limited therapeutic efficacy. Unlike Embryonic Stem Cells (ESCs), MSCs are considered adult stem cells, and have a limited proliferative capacity. When cultured *in vitro*, they age, affecting their therapeutic properties, particularly after long-term culture (199). As very large numbers of MSCs are required for therapeutic applications, *in vitro* expansion is required.

Overall, MSC treatment is mostly safe and well tolerated, however some MSC-related adverse events have still been recorded, being the most predominant fever (201), chills (196), headache (202), allergic rash, and liver dysfunction (203). Nevertheless, in both MSC-treated patients and controls, adverse events were recorded, indicating that the side effects might be infusion-related (195, 204). Moreover, the baseline status of patients with co-existing illnesses and the speed of infusion may be associated with the appearance of adverse events (195).

Treatment with MSCs has several benefits (205) such as: a) can quickly expand to a clinical volume at the appropriate time, b) allogeneic mesenchymal stem cells have not yet been associated with any negative side effects in clinical trials using mesenchymal cells, c) feasible storage for future therapeutic use, d) mesenchymal stem cells are multipotent stem cells, e) suitability and efficacy of mesenchymal stem cells have been known in many clinical trials, f) easy availability as can be obtained from various tissues such as adipose tissues and bone marrow (205).

### 4.5 Vaccination

While the development of vaccines has taken typically around 8 to 15 years, the current development of multiple vaccines has been unraveled in less than 12 months (206). The remarkable effectiveness of vaccines against SARS-CoV-2, particularly 95% for BNT162b2 (BioNTech/Pfizer) and 94.1% for mRNA-1273 (Moderna), has already been discussed and described extensively (207, 208). Numerous studies have shown that natural immunity seems to persist for a reasonable amount of time (209–211). According to epidemiological research, natural immunity provides protection against reinfections for longer than a year with little, if any, deterioration throughout this period (212–216). The majority of patients’ persistence of anti-SARS-CoV-2 antibodies and cellular immunity over more than a year supports the idea of long-term protection against reinfections. It is worth mentioning that some studies indicate that protection against reinfection reaches its highest level around 4 to 5 months following the initial infection, and then gradually declines. This observation could potentially be attributed to persistent viral shedding or the misclassification of prolonged SARS-CoV-2 infections as reinfections (215–217). In comparison to those who get two doses of an mRNA vaccine, observational studies suggest that natural immunity provides equivalent or higher protection against SARS-CoV-2 infections. However, the data are not entirely consistent, since the loss of natural immunity appears to be very moderate, but there is substantial evidence supporting the notion that protection against SARS-CoV-2 infections conferred by vaccination, tends to diminish relatively quickly over time (218–220). People who have previously contracted SARS-CoV-2 but were afterwards immunized against it, or vice versa, are said to have “hybrid immunity” (219–222). Although the data from the SARS-CoV-2 vaccine randomized controlled trials (RCTs) on hybrid immunity is conflicting, it does appear to be superior to either vaccine-induced (without a booster) or natural immunity alone. Hybrid immunization suggests a higher level of protection when infection and subsequent immunization occur at least 6 months apart as opposed to a shorter gap (223).

Low vaccination rates in Africa, with a large number of countries with less than 10% of the population with vaccination coverage, the lifting or end of restrictions in much of Western countries, the possible appearance of new variants (as it is happening with BQ1.1XBB variants with higher viral scape to previous immunity) and the burst of cases in Asia, which is experiencing the worst situation since the beginning of 2020, is a cause for concern. Therefore, the development of novel alternative treatments is mandatory.

## 5 Rationale for use of MSC and combinatorial cell therapy in severe to critical COVID-19 patients

In both animal models and human therapeutic trials, MSC therapy inhibited the aberrant immune-mediated inflammatory response brought on by influenza virus infection (1, 224). There have been several clinical studies for stem cell treatment since the outbreak of the COVID-19 pandemic and quite a few have shown that MSCs not only lead to noteworthy reduction of lung damage and recovery time, but also increased patient survival and early-stage tolerance (173, 201, 203, 204).

For patients with high mortality risk from COVID-19, a combination of safe MSC and memory T-cells specific for SARS-CoV-2 (CD45RA-) might be the best course of action. CD45RA- will provide the anti-viral effect during the first days, while MSCs will inhibit the complications of the second phase.

## 6 Conclusion

MSCs have important immunoregulatory effects on inflammation. Since the effects of SARS-CoV-2 on patients’ bodies are widespread, wherein both the release of pro-inflammatory cytokines, severe damage of alveolar epithelial cells (225) together with systemic effects suggest that the use of MSC might be beneficial in severe-to-critically ill patients, promoting their recovery period and as a result of their build-up in the lung capillary network and repairing the damaged tissue (226). Furthermore, the lack of TMPRSS2 and ACE2 receptors on the MSC makes them resistant to SARS-CoV-2 infection.

Even more, the preliminary proof demonstrating the feasibility and safety of treating COVID-19 patients with moderate to severe symptoms using convalescent CD45RA- memory T-cells, and due to the fact that memory T-cells can swiftly tackle the infection and offer ongoing immune support to lessen the severity of the COVID-19 symptoms, this strategy might eradicate virally infected cells and provide T-cell immunity to prevent recurrent reinfections. Finally, Baricitinib and dexamethasone have proven effective while reducing inflammation and effectively treat COVID-19 patients who are hospitalized (227) however, Baricitinib had less side effects than dexamethasone, and this will help clinicians choose which immunomodulatory medication to administer to patients based on their particular risks for dexamethasone-related side effects (228).

Despite existence of antivirals and neutralizing antibodies specifically developed for SARS-CoV-2 with good results in clinical trials, there is still a huge margin of improvement: problems such as new variants of concern which scape to antibodies or appearance of resistance to antivirals (as already described for Paxlovid) demand the development of new treatments as well as focusing in efficient combinational therapies that show better treatment outcomes when administered together.

## Author contributions

BS Conception and design, collection and assembly of data, data analysis and interpretation, manuscript writing and editing and financial support. BS, AG, EA, LH-B and CS: Collection and assembly of data, data analysis and interpretation, write the first draft. BS-J: writing the first draft of the clinical trial; CS, AM-Q, AP-M and SQ: reviewed the first version. All authors are members of the research team of the DECODE project (ICI21/0016: It is mandatory to decrease COVID-19 deaths). All authors contributed to the article and approved the submitted version.
